# Supplementary material for: Chronic kidney disease and the outcomes of fibrinolysis for ST-segment elevation myocardial infarction: A real-world study
Source: PLoS One. 2021 Jan 19;16(1):e0245576. doi: 10.1371/journal.pone.0245576 (PMC7815111; doi:10.1371/journal.pone.0245576)
Supplement: S1 Table — (DOCX) [file pone.0245576.s001.docx]

**S1 Table. Crude incidence rates of short-term outcomes by whether received fibrinolytic therapy among patients with and without chronic kidney disease (eGFR <60 mL/min/1.73 m^2^).**

|  | eGFR ≥60 mL/min/1.73 m^2^ (n=8226) | | eGFR <60 mL/min/1.73 m^2^ (n=1282) | |
| --- | --- | --- | --- | --- |
|  | No fibrinolysis (n=5025) | Fibrinolysis (n=3201) | No fibrinolysis (n=944) | Fibrinolysis (n=338) |
| MACEs (%) | 315 (6.3) | 149 (4.7) | 177 (18.8) | 75 (22.2) |
| All-cause mortality (%) | 295 (5.9) | 133 (4.2) | 169 (17.9) | 74 (21.9) |
| Recurrent MI (%) | 21 (0.4) | 20 (0.6) | 7 (0.7) | 5 (1.5) |
| Stroke (%) | 7 (0.1) | 3 (0.1) | 5 (0.5) | 0 (0.0) |
| Severe bleeding (%) | 22 (0.4) | 24 (0.8) | 12 (1.3) | 9 (2.7) |

The results are presented as n (%).

eGFR, estimated glomerular filtration rate; MACEs, major adverse cardiovascular events; MI, myocardial infarction.
