# Supplementary material for: Chronic kidney disease and the outcomes of fibrinolysis for ST-segment elevation myocardial infarction: A real-world study
Source: PLoS One. 2021 Jan 19;16(1):e0245576. doi: 10.1371/journal.pone.0245576 (PMC7815111; doi:10.1371/journal.pone.0245576)
Supplement: S4 Table — (DOCX) [file pone.0245576.s004.docx]

**S4 Table. Baseline characteristics of propensity score-matched subgroup**

|  | eGFR ≥60 mL/min/1.73 m^2^ (n=5502) | | | eGFR <60 mL/min/1.73 m^2^ (n=588) | | |
| --- | --- | --- | --- | --- | --- | --- |
|  | No fibrinolysis (n=2751) | Fibrinolysis (n=2751) | *P* for difference^*^ | No fibrinolysis (n=294) | Fibrinolysis (n=294) | *P* for difference^*^ |
| Age (years) | 60.9±12.3 | 60.9±10.5 | 0.916 | 68.2±11.2 | 67.6±10.1 | 0.475 |
| Men (%) | 2072 (75.3) | 2076 (75.5) | 0.900 | 185 (62.9) | 188 (64.0) | 0.797 |
| eGFR (mL/min/1.73 m^2^) | 105 (86–129) | 104 (84–129) | 0.422 | 49 (40–56) | 50 (39–55) | 0.675 |
| Hospital stay (days) | 11 (6–13) | 11 (7–14) | 0.601 | 8 (3–13) | 8 (2–13) | 0.924 |
| Delay to admission (%) | 1527 (55.5) | 1521 (55.3) | 0.871 | 174 (59.2) | 175 (59.5) | 0.933 |
| Education ≥High school (%) | 336 (12.2) | 342 (12.4) | 0.806 | 32 (10.9) | 38 (12.9) | 0.445 |
| Farmer (%) | 1786 (64.9) | 1783 (64.8) | 0.933 | 182 (61.9) | 180 (61.2) | 0.865 |
| Current smoking (%) | 1057 (38.4) | 1084 (39.4) | 0.455 | 63 (21.4) | 68 (23.1) | 0.620 |
| History of disease (%) |  |  |  |  |  |  |
| Hypertension | 1710 (62.2) | 1725 (62.7) | 0.676 | 183 (62.2) | 190 (64.6) | 0.549 |
| Diabetes | 303 (11.0) | 299 (10.9) | 0.863 | 45 (15.3) | 45 (15.3) | 1.000 |
| Myocardial infarction | 137 (5.0) | 130 (4.7) | 0.661 | 15 (5.1) | 18 (6.1) | 0.591 |
| Angina | 266 (9.7) | 275 (10.0) | 0.684 | 22 (7.5) | 29 (9.9) | 0.305 |
| Heart Failure | 26 (1.0) | 31 (1.1) | 0.506 | 8 (2.7) | 10 (3.4) | 0.632 |
| Stroke | 214 (7.8) | 212 (7.7) | 0.920 | 23 (7.8) | 28 (9.5) | 0.464 |
| SBP <90 mmHg (%) | 109 (4.0) | 114 (4.1) | 0.733 | 48 (16.3) | 47 (16.0) | 0.911 |
| Heart rate ≥100 beats/m (%) | 204 (7.4) | 220 (8.0) | 0.419 | 54 (18.4) | 45 (15.3) | 0.321 |
| Continuous ECG monitoring (%) | 2645 (96.2) | 2649 (96.3) | 0.777 | 287 (97.6) | 285 (96.9) | 0.612 |
| In-hospital medication taken (%) |  |  |  |  |  |  |
| Aspirin | 2727 (99.1) | 2732 (99.3) | 0.444 | 285 (96.9) | 285 (96.9) | 1.000 |
| Clopidogrel | 2581 (93.8) | 2576 (93.6) | 0.781 | 274 (93.2) | 268 (91.2) | 0.357 |
| ACEI/ARB | 1633 (59.4) | 1649 (59.9) | 0.660 | 155 (52.7) | 149 (50.7) | 0.621 |
| β-Blockers | 1872 (68.1) | 1893 (68.8) | 0.543 | 166 (56.5) | 162 (55.1) | 0.740 |
| CCB | 147 (5.3) | 166 (6.0) | 0.269 | 28 (9.5) | 25 (8.5) | 0.666 |
| Statins | 2654 (96.5) | 2659 (96.7) | 0.711 | 276 (93.9) | 269 (91.5) | 0.268 |
| QCI intervention (%) | 1285 (46.7) | 1280 (46.5) | 0.893 | 134 (45.6) | 137 (46.6) | 0.804 |
| Intervention cycle (%) |  |  |  |  |  |  |
| 1 | 584 (21.2) | 599 (21.8) | 0.984 | 56 (19.1) | 59 (20.1) | 0.983 |
| 2 | 535 (19.5) | 534 (19.4) |  | 60 (20.4) | 56 (19.1) |  |
| 3 | 549 (20.0) | 546 (19.9) |  | 55 (18.7) | 54 (18.4) |  |
| 4 | 566 (20.6) | 552 (20.1) |  | 67 (22.8) | 65 (22.1) |  |
| 5 | 517 (18.8) | 520 (18.9) |  | 56 (19.1) | 60 (20.4) |  |

The results are presented as mean ± SD, median (quartile 1–quartile 3), or n (%).

^*^Calculated by using a t test, Wilcoxon rank test, or chi-square test.

eGFR, estimated glomerular filtration rate; SBP, systolic blood pressure; ECG, electrocardiograph; ACEI, angiotensin-converting enzyme inhibitors; ARB, angiotensin receptor blockers; CCB, calcium channel blockers; QCI, quality of care initiatives.
