# Supplementary material for: Chronic kidney disease and the outcomes of fibrinolysis for ST-segment elevation myocardial infarction: A real-world study
Source: PLoS One. 2021 Jan 19;16(1):e0245576. doi: 10.1371/journal.pone.0245576 (PMC7815111; doi:10.1371/journal.pone.0245576)
Supplement: S5 Table — (DOCX) [file pone.0245576.s005.docx]

**S5 Table. Crude incidence rates of short-term outcomes by whether received fibrinolytic therapy among patients with and without chronic kidney disease (eGFR <60 mL/min/1.73 m^2^), results of propensity score-matched subgroup**

|  | eGFR ≥60 mL/min/1.73 m^2^ (n=5502) | | eGFR <60 mL/min/1.73 m^2^ (n=588) | |
| --- | --- | --- | --- | --- |
|  | No fibrinolysis (n=2751) | Fibrinolysis (n=2751) | No fibrinolysis (n=294) | Fibrinolysis (n=294) |
| MACEs (%) | 140 (5.1) | 143 (5.2) | 49 (16.7) | 66 (22.5) |
| All-cause mortality (%) | 132 (4.8) | 127 (4.6) | 45 (15.3) | 65 (22.1) |
| Recurrent MI (%) | 11 (0.4) | 18 (0.7) | 3 (1.0) | 4 (1.4) |
| Stroke (%) | 1 (0.0) | 3 (0.1) | 2 (0.7) | 0 (0.0) |
| Severe bleeding (%) | 10 (0.4) | 22 (0.8) | 2 (0.7) | 8 (2.7) |

The results are presented as n (%).

eGFR, estimated glomerular filtration rate; MACEs, major adverse cardiovascular events; MI, myocardial infarction.
