# Supplementary material for: Chronic kidney disease and the outcomes of fibrinolysis for ST-segment elevation myocardial infarction: A real-world study
Source: PLoS One. 2021 Jan 19;16(1):e0245576. doi: 10.1371/journal.pone.0245576 (PMC7815111; doi:10.1371/journal.pone.0245576)
Supplement: S6 Table — (DOCX) [file pone.0245576.s006.docx]

**S6 Table. Associations between fibrinolytic therapy and the risk of short-term major adverse cardiovascular events among patients with and without chronic kidney disease (eGFR <60 mL/min/1.73 m^2^), results of propensity score-matched subgroup**

|  | eGFR ≥60 mL/min/1.73 m^2^ (n=5502) | | eGFR <60 mL/min/1.73 m^2^ (n=588) | | *P* for interaction |
| --- | --- | --- | --- | --- | --- |
|  | RR (95% CI) | *P* value | RR (95% CI) | *P* value |  |
| Model 1^*^ |  |  |  |  |  |
| No fibrinolysis | Ref | / | Ref | / | / |
| Fibrinolysis | 0.80 (0.60 to 1.06) | 0.126 | 1.02 (0.69 to 1.51) | 0.920 | 0.158 |
| Model 2^†^ |  |  |  |  |  |
| No fibrinolysis | Ref | / | Ref | / |  |
| Fibrinolysis | 0.78 (0.58 to 1.04) | 0.092 | 0.99 (0.67 to 1.47) | 0.978 | 0.152 |

^*^Adjusted for age, sex, intervention, cycle, fibrin-specific thrombolytic agent, delay to admission, and delay to fibrinolytic therapy.

^†^Further adjusted for propensity scores.

eGFR, estimated glomerular filtration rate; RR, relative risk; CI, confidence interval.
