# Supplementary material for: Chronic kidney disease and the outcomes of fibrinolysis for ST-segment elevation myocardial infarction: A real-world study
Source: PLoS One. 2021 Jan 19;16(1):e0245576. doi: 10.1371/journal.pone.0245576 (PMC7815111; doi:10.1371/journal.pone.0245576)
Supplement: S7 Table — (DOCX) [file pone.0245576.s007.docx]

**S7 Table. Crude incidence rates of short-term outcomes by no, failed, and successful fibrinolysis, among patients with and without chronic kidney disease (eGFR <60 mL/min/1.73 m^2^), results of propensity score-matched subgroup**

|  | eGFR ≥60 mL/min/1.73 m^2^ (n=5502) | | | eGFR <60 mL/min/1.73 m^2^ (n=588) | | |
| --- | --- | --- | --- | --- | --- | --- |
|  | No fibrinolysis (n=2751) | Successful fibrinolysis (n=2259) | Failed fibrinolysis (n=492) | No fibrinolysis (n=294) | Successful fibrinolysis (n=196) | Failed fibrinolysis (n=98) |
| MACEs (%) | 140 (5.1) | 66 (2.9) | 77 (15.7) | 49 (16.7) | 20 (10.2) | 46 (46.9) |
| All-cause mortality (%) | 132 (4.8) | 53 (2.4) | 74 (15.0) | 45 (15.3) | 19 (9.7) | 46 (46.9) |
| Recurrent MI (%) | 11 (0.4) | 13 (0.6) | 5 (1.0) | 3 (1.0) | 2 (1.0) | 2 (2.0) |
| Stroke (%) | 1 (0.0) | 2 (0.1) | 1 (0.2) | 2 (0.7) | 0 (0.0) | 0 (0.0) |
| Severe bleeding (%) | 10 (0.4) | 17 (0.8) | 5 (1.0) | 2 (0.7) | 3 (1.5) | 5 (5.1) |

The results are presented as n (%).

eGFR, estimated glomerular filtration rate; MACEs, major adverse cardiovascular events; MI, myocardial infarction.
