# Supplementary material for: Chronic kidney disease and the outcomes of fibrinolysis for ST-segment elevation myocardial infarction: A real-world study
Source: PLoS One. 2021 Jan 19;16(1):e0245576. doi: 10.1371/journal.pone.0245576 (PMC7815111; doi:10.1371/journal.pone.0245576)
Supplement: S8 Table — (DOCX) [file pone.0245576.s008.docx]

**S8 Table. Associations of fibrinolytic therapy with risk of short-term major adverse cardiovascular events among patients with and without chronic kidney disease (eGFR <60 mL/min/1.73 m^2^), results of propensity score-matched subgroup**

|  | eGFR ≥60 mL/min/1.73 m^2^ (n=5502) | | eGFR <60 mL/min/1.73 m^2^ (n=588) | | *P* for interaction |
| --- | --- | --- | --- | --- | --- |
|  | RR (95% CI) | *P* value | RR (95% CI) | *P* value |  |
| Model 1^*^ |  |  |  |  |  |
| No fibrinolysis | Ref | / | Ref | / | / |
| Successful fibrinolysis | 0.41 (0.27 to 0.63) | <0.001 | 0.50 (0.27 to 0.93) | 0.028 | 0.474 |
| Failed fibrinolysis | 2.15 (1.51 to 3.07) | <0.001 | 2.02 (1.33 to 3.07) | 0.001 | 0.765 |
| Model 2^†^ |  |  |  |  |  |
| No fibrinolysis | Ref | / | Ref | / | / |
| Successful fibrinolysis | 0.40 (0.26 to 0.62) | <0.001 | 0.49 (0.27 to 0.90) | 0.023 | 0.462 |
| Failed fibrinolysis | 2.10 (1.48 to 2.98) | <0.001 | 1.98 (1.30 to 3.02) | 0.002 | 0.794 |

^*^Adjusted for age, sex, intervention, cycle, fibrin-specific thrombolytic agent, delay to admission, and delay to fibrinolytic therapy.

^†^Further adjusted for propensity scores.

eGFR, estimated glomerular filtration rate; RR, relative risk; CI, confidence interval.
