# Supplementary material for: Chronic kidney disease and the outcomes of fibrinolysis for ST-segment elevation myocardial infarction: A real-world study
Source: PLoS One. 2021 Jan 19;16(1):e0245576. doi: 10.1371/journal.pone.0245576 (PMC7815111; doi:10.1371/journal.pone.0245576)
Supplement: S10 Table — (DOCX) [file pone.0245576.s010.docx]

**S10 Table. Associations of fibrinolytic therapy with risk of short-term major adverse cardiovascular events among patients with and without chronic kidney disease (eGFR <60 mL/min/1.73 m^2^), sensitivity analysis excluding 1565 participants with SBP <90 mmHg and/or heart rate ≥100 beats/min**

|  | eGFR ≥60 mL/min/1.73 m^2^ (n=7088) | | eGFR <60 mL/min/1.73 m^2^ (n=855) | | *P* for interaction |
| --- | --- | --- | --- | --- | --- |
|  | RR (95% CI) | *P* value | RR (95% CI) | *P* value |  |
| Model 1^*^ |  |  |  |  |  |
| No fibrinolysis | Ref | / | Ref | / | / |
| Successful fibrinolysis | 0.45 (0.30 to 0.69) | <0.001 | 0.59 (0.32 to 1.12) | 0.106 | 0.480 |
| Failed fibrinolysis | 1.67 (1.18 to 2.36) | 0.004 | 2.17 (1.53 to 3.09) | <0.001 | 0.298 |
| Model 2^†^ |  |  |  |  |  |
| No fibrinolysis | Ref | / | Ref | / | / |
| Successful fibrinolysis | 0.70 (0.57 to 0.86) | <0.001 | 0.80 (0.57 to 1.11) | 0.175 | 0.504 |
| Failed fibrinolysis | 1.28 (1.08 to 1.51) | 0.003 | 1.48 (1.24 to 1.76) | <0.001 | 0.240 |

^*^Adjusted for age, sex, intervention, cycle, fibrin-specific thrombolytic agent, delay to admission, and delay to fibrinolytic therapy.

^†^Further adjusted for eGFR, education, occupation, current smoking, body mass index, history of diabetes mellitus, hypertension, cardiovascular disease, systolic blood pressure lower than 90 mmHg when presenting at hospital, heart rate higher than 100 beats/m when presenting at hospital, in-hospital use of aspirin, clopidogrel, angiotensin-converting enzyme inhibitors/angiotensin receptor blockers, β-Blockers, calcium channel blockers, and statins.

eGFR, estimated glomerular filtration rate; RR, relative risk; CI, confidence interval.
