# Supplementary material for: Chronic kidney disease and the outcomes of fibrinolysis for ST-segment elevation myocardial infarction: A real-world study
Source: PLoS One. 2021 Jan 19;16(1):e0245576. doi: 10.1371/journal.pone.0245576 (PMC7815111; doi:10.1371/journal.pone.0245576)
Supplement: S11 Table — (DOCX) [file pone.0245576.s011.docx]

**S11 Table. Comparison of baseline characteristics and outcomes between participants included (n=9508) and excluded because of missing data on serum creatinine concentration (n=768)**

|  | Included (n=9508) | Excluded (n=786) | *P* for difference^*^ |
| --- | --- | --- | --- |
| Age (years) | 64.0±12.4 | 63.7±12.9 | 0.542 |
| Hospital stay (days) | 10 (6–14) | 3 (0–11) | <0.001 |
| Men (%) | 6664 (70.1) | 544 (69.2) | 0.606 |
| Education ≥High school (%) | 1049 (11.0) | 64 (8.1) | 0.012 |
| Farmer (%) | 6259 (65.8) | 477 (60.7) | 0.004 |
| Current smoking (%) | 3135 (33.0) | 203 (25.8) | <0.001 |
| History of disease (%) |  |  |  |
| Hypertension | 5976 (62.9) | 428 (54.5) | <0.001 |
| Diabetes | 1103 (11.6) | 92 (11.7) | 0.930 |
| Myocardial infarction | 570 (6.0) | 46 (5.9) | 0.871 |
| Angina | 1034 (10.9) | 64 (8.1) | 0.017 |
| Heart Failure | 249 (2.6) | 12 (1.5) | 0.061 |
| Stroke | 834 (8.8) | 42 (5.3) | <0.001 |
| SBP <90 mmHg (%) | 446 (4.7) | 70 (8.9) | <0.001 |
| Heart rate ≥100 beats/m (%) | 1183 (12.4) | 93 (11.8) | 0.618 |
| Continuous ECG monitoring (%) | 8662 (91.1) | 614 (78.1) | <0.001 |
| Fibrinolytic therapy (%) | 3539 (37.2) | 222 (28.2) | <0.001 |
| In-hospital medication taken (%) |  |  |  |
| Aspirin | 9267 (97.5) | 723 (92.1) | <0.001 |
| Clopidogrel | 8402 (88.4) | 583 (74.2) | <0.001 |
| ACEI/ARB | 5571 (58.6) | 329 (41.9) | <0.001 |
| β-Blockers | 6165 (64.8) | 353 (44.9) | <0.001 |
| CCB | 705 (7.4) | 37 (4.7) | 0.005 |
| Statins | 8960 (94.2) | 636 (80.9) | <0.001 |
| QCI intervention (%) | 4513 (47.5) | 398 (50.6) | 0.087 |
| Intervention cycle (%) |  |  |  |
| 1 | 2034 (21.4) | 165 (21.0) | 0.509 |
| 2 | 1823 (19.2) | 151 (19.2) |  |
| 3 | 1944 (20.5) | 171 (21.8) |  |
| 4 | 1925 (20.3) | 141 (17.8) |  |
| 5 | 1782 (18.7) | 158 (20.1) |  |
| Short-term MACEs | 716 (7.5) | 166 (21.2) | <0.001 |
| All-cause death | 671 (7.1) | 162 (20.6) | <0.001 |

The results are presented as mean ± SD, median (quartile 1–quartile 3), or n (%).

^*^Calculated by using a t test, Wilcoxon rank test, or chi-square test.

SBP, systolic blood pressure; ECG, electrocardiograph; ACEI, angiotensin-converting enzyme inhibitors; ARB, angiotensin receptor blockers; CCB, calcium channel blockers; QCI, quality of care initiatives; MACEs, major adverse cardiovascular events.
