# Supplementary material for: Chronic kidney disease and the outcomes of fibrinolysis for ST-segment elevation myocardial infarction: A real-world study
Source: PLoS One. 2021 Jan 19;16(1):e0245576. doi: 10.1371/journal.pone.0245576 (PMC7815111; doi:10.1371/journal.pone.0245576)
Supplement: Attachment — Submitted filename: Response to Reviewers-2020-11-11.docx [file pone.0245576.s013.docx]

**Re：Reduced kidney function and the outcomes of fibrinolysis for ST-segment elevation myocardial infarction: A real-world study**

**(PONE-D-20-21065)**

**PLOS ONE**

**Response to Reviewer #1**

**Comment 1:**

This is an interesting observational analysis from an established network, the Clinical Pathways in Acute Coronary Syndromes program (CPACS-3), a large study of the management of suspected acute coronary syndromes (ACS) in 101 county hospitals without onsite PCI capabilities in China.

The study is quite well presented, analyzed, and interpreted. Fibrinolysis remains an important reperfusion modality for many patients worldwide and these findings may have relevant implications for their management.

**Response:**

Thanks for your comments.

**Change in the manuscript:**

No change.

**Comment 2:**

I have some suggestions for the author to consider:

MAJOR

For this research question it may be relevant to specify when the diagnosis of renal insufficiency was performed in relationship to the decision to perform fibrinolysis. Specifically, distinguish patients with known renal insufficiency at the time of fibrinolysis (such as patients on hemodialysis), patients with a new diagnosis of renal insufficiency when creatinine was collected (immediately after the fibrinolysis I presume), from patients without renal insufficiency. This may help better understand the results and if, for patients with unknown renal function status, this information should be waited for before deciding fibrinolysis.

**Response:**

Accepted. In this study, creatinine was measured at the admission. Besides, we added a paragraph to further discuss our results according to your suggestions.

**Change in the manuscript:**

1. We clearly indicated creatinine was measured at the admission (**Page 3, Lines 16 to 17, and Page 9, Line 19**).

2. A paragraph has been added in the revised manuscript (**Page 18, Lines 4 to 7**): “A fibrin-specific thrombolytic agent should be the priority selection for CKD patients accepting fibrinolytic therapy. Therefore, clinicians should distinguish patients with known renal insufficiency at the time of fibrinolysis, from patients without renal insufficiency before deciding fibrinolysis”.

**Comment 3:**

The discussion is well structured in general but might be improved by:

Shortly speculating as to why an increased failure of fibrinolysis is observed in patients with RKF. Hypofibrinolysis for example has been well documented in patients with renal insufficiency and end-stage renal disease and could be mentioned, and the potential implications for different agents/dosage in this setting.

**Response:**

Thank you so much for this valuable comment. We added several sentences in the revised manuscript to describe the underlying mechanisms of higher failure rate of fibrinolysis in patient with CKD.

**Change in the manuscript:**

Sentences have been added in the revised manuscript to describe the underlying mechanisms of higher failure rate of fibrinolysis in patient with CKD (**Page 18, Lines 1 to 10**): “Several underlying mechanisms may account for that CKD patients had a higher rate of failed fibrinolysis. Firstly, hypofibrinolysis has been well documented in patients with renal insufficiency and end-stage renal disease, which might lead to the higher rate of failed fibrinolysis. A fibrin-specific thrombolytic agent should be the priority selection for CKD patients accepting fibrinolytic therapy. Therefore, clinicians should distinguish patients with known renal insufficiency at the time of fibrinolysis, from patients without renal insufficiency before deciding fibrinolysis. Secondly, CKD patients had more diffuse coronary artery disease with a higher thrombotic burden than non-CKD patients. Thirdly, patients with CKD could have more risk factors, such as diabetes, hypertension, and obesity, which might result in failed fibrinolysis”.

**Comment 4:**

What are the authors suggestions for managing these patients in terms of diagnosis (for eg. should we routinely wait creatinine results before performing fibrinolyis ?) and treatments (eg. considerations to accept a delay higher than 120 minutes to urgent angiography and primary PCI in these patients?)

**Response:**

Thanks again. According to your suggestion, we added a paragraph to indicate our suggestions for managing patients with CKD in terms of diagnosis and treatments after STEMI.

**Change in the manuscript:**

We added a sentence to indicate our suggestions for managing patients with CKD in terms of diagnosis and treatments after STEMI (**Page 19, Lines 16 to 18**): “Kidney function should be evaluated before performing fibrinolysis, and a fibrin-specific thrombolytic agent should be the priority selection for CKD patients when PCI is not available”.

**Comment 5:**

MINOR

Reduced kidney function, RKF, is not a standard acronym. The authors may consider using chronic kidney disease or KDIGO equivalents.

**Response:**

Thank you for your suggestion. The term “reduced kidney function, RKF” has been modified to “chronic kidney disease, CKD” throughout this manuscript.

**Change in the manuscript:**

The term “reduced kidney function, RKF” has been modified to “chronic kidney disease, CKD” throughout this manuscript.

**Comment 6:**

www.clinicaltrails.gov under Trial registration is misspelled.

**Response:**

We are sorry for this mistake.

**Change in the manuscript:**

Done (**Page 4, Line 16 and Page 9, Line 6**).

**Comment 7:**

Page 11, line 189: “The secondary outcome was all-cause mortality, recurrent MI, stroke, and severe bleeding”. The logic here, as for every composite and correctly reported for the primary endpoint is OR, that is “The secondary outcome was all-cause mortality, recurrent MI, stroke, OR severe bleeding”.

**Response:**

Accepted.

**Change in the manuscript:**

The change has been made (**Page 3, Lines 17 to 18 and** **Page 10, Line 22 to Page 11, Line 1**).

**Response to Reviewer #2**

**Comment 1:**

In this study the authors analyzed the data from a multicentric registry that involved a considerable number of acute coronary syndrome patients admitted to 101 country hospital in China without the capability to perform primary PCI.

The aim of the study was to assess whether reduced kidney function (RKF) may modify the association between fibrinolytic therapy and in-hospital major adverse cardiovascular events (MACE) – the primary endpoint.

The key results are the following:

1) patients with RKF were more likely to experience in-hospital MACEs compared to those with normal kidney function (19.7% vs 5.6%, respectively)

2) fibrinolytic therapy was associated with a 13% reduction in the primary endpoint in patients with normal kidney function (RR 0.87, 95% CI 0.76-0.99), but not in the those with RKF (RR 1.02, 95% CI 0.87-1.18)

3) Failed fibrinolytic therapy was higher in RFK patients compared to those with normal kidney function (32.8% vs 16.9%)

4) Failed fibrinolysis was not independently associated with the end-point at multivariate analysis (page 14, lines 259-261).

In the discussion (page 17, lines 324-325), the authors comment these results as follows: “in the current study, we found that successful fibrinolysis occurred less frequently in patients with RKF, likely contributing to a higher risk of in-hospital MACE”. The same thought is replicated in the conclusion of the abstract (“RFK modified the association of fibrinolytic therapy with the risk of in-hospital mortality via its negative association with successful fibrinolysis”, page 4, lines 64-65). However, this perspective seems to be contradicted by the results of multivariate analysis showing no independent association between adverse events and failed fibrinolysis and by the fact that in the subgroup with RKF fibrinolytic therapy was not associated with lower incidence of MACE. A possible explanation is that fibrinolytic therapy improves the prognosis in patients with normal kidney function but not in the RKF population (see point number 2) because the latter group is fragile and at-risk of adverse events independently from successful coronary reperfusion.

**Response:**

Thanks for your comments. We consider this perspective is not contradicted by the results that fibrinolytic therapy was not associated with lower incidence of MACE in patients with CKD. Actually, we found that failed fibrinolysis was associated with a similarly higher risk of in-hospital MACEs in both CKD and non-CKD patients. In short, CKD patients had a higher rate of failed fibrinolysis than non-CKD patients after STEMI, leading to CKD patients had a higher rate of in-hospital MACEs. Besides, stratified analyses showed that successful fibrinolysis was associated with a similarly lower risk among patients with and without CKD, while failed fibrinolysis was associated with a similarly higher risk in both CKD and non-CKD patients. Therefore, we consider that the higher rate of failed fibrinolysis in CKD patients is the key reason why CKD modified the association between fibrinolysis and in-hospital MACEs.

**Change in the manuscript:**

No change.

**Comment 2:**

In my opinion, the most important finding of the study is that patients with RKF have a lower rate of successful fibrinolytic therapy and the authors should speculate in the discussion what led to this significant result (more diffuse coronary artery disease with a higher thrombotic burden in RKF patients?) and to its clinical implications (preference for ptca?).

**Response:**

Accepted. Yes, the key point of this study is that patients with CKD had a lower success rate of fibrinolysis, the later in turn led to a significant higher risk of in-hospital MACEs. We add a paragraph to discuss the underlying mechanisms of higher failure rate of fibrinolysis in patient with CKD and its clinical implications.

**Change in the manuscript:**

The following sentences have been added in the revised manuscript (**Page 18, Lines 1 to 10**): “Several underlying mechanisms may account for the higher rate of failed fibrinolysis. Firstly, hypofibrinolysis has been well documented in patients with renal insufficiency and end-stage renal disease, which might lead to the higher rate of failed fibrinolysis. A fibrin-specific thrombolytic agent should be the priority selection for CKD patients accepting fibrinolytic therapy. Therefore, clinicians should distinguish patients with known renal insufficiency at the time of fibrinolysis, from patients without renal insufficiency before deciding fibrinolysis. Secondly, CKD patients had more diffuse coronary artery disease with a higher thrombotic burden than non-CKD patients. Thirdly, CKD was associated with many of risk factors, such as diabetes, hypertension, and obesity, which might result in failed fibrinolysis”.

**Comment 4:**

Lastly, I am in disagreement with the inclusion in the primary endpoint (“in-hospital MACEs”) of patients who died within 1 week after a discharge against medical advice, under the category of all-cause mortality. Apart from being a contradiction itself, it introduces a significant bias since this entity cannot be measured. **Response:**

Thanks for this comment. Yes, we agree with you that including those patients who died within 1 week after a discharge against medical advice might lead to bias of measuring outcome. However, if we did not include these out-of-hospital deaths, the bias would be greater, as Chinese people would prefer to die at home after relinquishing curative treatments.

**Change in the manuscript:**

No change.
