# Supplementary material for: Chronic kidney disease and the outcomes of fibrinolysis for ST-segment elevation myocardial infarction: A real-world study
Source: PLoS One. 2021 Jan 19;16(1):e0245576. doi: 10.1371/journal.pone.0245576 (PMC7815111; doi:10.1371/journal.pone.0245576)
Supplement: Attachment — Submitted filename: Response to Reviewers-2021-01-04.docx [file pone.0245576.s014.docx]

**Re：Chronic kidney disease and the outcomes of fibrinolysis for ST-segment elevation myocardial infarction: A real-world study**

**(PONE-D-20-21065R1)**

**PLOS ONE**

**Response to Reviewer #1**

**Comment 1:**

The authors have mostly and completely included my comments.

The wording of some sentences, such as the conclusions, may be improved. CKD is indeed and chronic and pre-existing conditions, not a complication. Why don't simply say something such as: "CKD reduced the likelihood of successful fibrinolysis and increased the risk of in-hospital MACEs in patients with STEMI (...)".

**Response:**

Thanks for your comments. We revised the conclusion and relevant sentences according to your suggestion (**Page 4, Lines 11 to 12; Page 19, Lines 14 to 15**).

**Response to Reviewer #2**

**Comment 1:**

Dear authors, thank-you for your reply. However, there is still one major point that in my opinion needs classification: in the Table 1, a multi variable analysis showed that failed fibrinolysis is not an independent predictor of mace in patients with CKD. In figure 1, another multi variable analysis including the same co-variates gave completely opposite results: this time it seems that CKD has no role in modifying the RR, that it only depends on successful fibrinolysis. I admit I am not a statistician, but I think you should help me and other readers to understand the reason of this.

**Response:**

Thanks for your comments. We have carefully checked the results in Table 1 and Figure 1. We found that Table 1 described the baseline characteristics of participants, so we suppose you actually saying is Table 2.

In Table 2, our results showed that “fibrinolysis” (**not failed fibrinolysis**) is an independent predictor of MACEs in patients without CKD (P=0.034), but not in those with CKD (P=0.846). The interaction test was significant (P=0.026).

In Figure 1, fibrinolysis was classified as successful and failed fibrinolysis. Our further analyses found that CKD did not modify the relationships of successful and failed fibrinolysis with MACEs.

To help the readers to better understand our article, we revised our main conclusion to “CKD reduced the likelihood of successful fibrinolysis and increased the risk of short-term MACEs in patients with STEMI” (**Page 4, Lines 11 to 12; Page 19, Lines 14 to 15**).

**Comment 2:**

Another minor comment: if you included mortality post-voluntary discharge because of a cultural habit (Chinese people often desire so), you should state it in the methods section and maybe modify “in-hospital MACE” with “short-term MACE”

**Response:**

Accepted. We added a sentence in the Methods section to state the reason why we included mortality post-voluntary discharge (**Page** **10, Lines 20 to 21**). Besides, “in-hospital MACEs” has been revised to “short-term MACEs” throughout this manuscript.
